# Characterization of the biotin uptake system encoded by the biotin-inducible *bioYMN *operon of *Corynebacterium glutamicum*

**DOI:** 10.1186/1471-2180-12-6

**Published:** 2012-01-13

**Authors:** Jens Schneider, Petra Peters-Wendisch, K Corinna Stansen, Susanne Götker, Stanislav Maximow, Reinhard Krämer, Volker F Wendisch

**Affiliations:** 1 Genetics of Prokaryotes, Faculty of Biology and CeBiTec, Bielefeld University, Bielefeld, Germany; 2 Institute for Biochemistry, Cologne University, Cologne, Germany; 3 Genetics of Prokaryotes, Bielefeld University, P.O. Box 100131, 33501 Bielefeld, Germany

## Abstract

**Background:**

The amino acid-producing Gram-positive *Corynebacterium glutamicum *is auxotrophic for biotin although biotin ring assembly starting from the precursor pimeloyl-CoA is still functional. It possesses AccBC, the α-subunit of the acyl-carboxylases involved in fatty acid and mycolic acid synthesis, and pyruvate carboxylase as the only biotin-containing proteins. Comparative genome analyses suggested that the putative transport system BioYMN encoded by *cg2147, cg2148 *and *cg2149 *might be involved in biotin uptake by *C. glutamicum*.

**Results:**

By comparison of global gene expression patterns of cells grown with limiting or excess supply of biotin or with dethiobiotin as supplement replacing biotin revealed that expression of genes coding for enzymes of biotin ring assembly and for the putative uptake system was regulated according to biotin availability. RT-PCR and 5'-RACE experiments demonstrated that the genes *bioY, bioM*, and *bioN* are transcribed from one promoter as a single transcript. Biochemical analyses revealed that BioYMN catalyzes the effective uptake of biotin with a concentration of 60 nM biotin supporting a half-maximal transport rate. Maximal biotin uptake rates were at least five fold higher in biotin-limited cells as compared to cells grown with excess biotin. Overexpression of *bioYMN *led to an at least 50 fold higher biotin uptake rate as compared to the empty vector control. Overproduction of BioYMN alleviated biotin limitation and interfered with triggering L-glutamate production by biotin limitation.

**Conclusions:**

The operon *bioYMN *from *C. glutamicum *was shown to be induced by biotin limitation. Transport assays with radio-labeled biotin revealed that BioYMN functions as a biotin uptake system. Overexpression of *bioYMN *affected L-glutamate production triggered by biotin limitation.

## Background

Biotin is a vitamin in humans (vitamin H or B7). Biotin deficiency is rarely observed in humans, e.g. after prolonged consumption of raw egg whites that contains biotin-binding avidin [1], as the normal microflora of the large intestine is considered to provide sufficient supply of biotin. If biotin is lacking, multiple carboxylase deficiencies arise [1] because biotin is a cofactor of the biotin-dependent carboxylases, which occur in all domains of life [2]. Many bacteria can synthesize biotin, but biotin auxotrophic bacteria such as *Corynebacterium glutamicum *require uptake of biotin from the habitat.

Biotin synthesis can be subdivided into synthesis of pimelic acid followed by the biotin ring assembly [3]. Biotin ring assembly occurs via the well-studied enzymes 8-amino-7-oxononanoate synthase, 7,8-diaminononanoate synthase, dethiobiotin synthase and biotin synthase encoded by *bioF, bioA, bioD *and *bioB*, respectively [2]. Pimelate synthesis occurs via two alternative routes as found in *Bacillus subtilis *and *Escherichia coli*, respectively [3]. In *B. subtilis*, pimeloyl-CoA is generated by interception of fatty acid biosynthesis by P450-dependent BioI, which yields pimeloyl-ACP chains by oxidative cleavage of long-chain acyl-ACPs [4]. In *E. coli*, malonyl-CoA methyl ester is generated by SAM-dependent methyltransferase BioC as a primer molecule and afterwards elongated in fatty acid biosynthesis to yield methyl-pimeloyl-ACP which finally is demethylated by carboxylesterase BioH [5]. Other sources of pimeloyl-CoA are externally added pimelic acid which is activated by pimeloyl-CoA synthetase as e.g. in *B. subtilis*, yet uncharacterized biosynthetic pathways as proposed e.g. for *Desulfovibrio *species [6] or degradation of benzene as e.g. in *Rhodopseudomonas palustris *[7].

*C. glutamicum *is a Gram-positive biotin-auxotrophic bacterium that was originally isolated as an L-glutamate producer from soil samples [8]. *C. glutamicum *lacks the ability to synthesize pimeloyl-CoA, but the enzymes for biotin ring assembly, BioA, BioD and BioB, are functional [9-11]. It has been proposed that biotin auxotrophy in *C. glutamicum *is due to the lack of a BioF homolog [9-11]. Accordingly, it has been found that biotin, dethiobiotin, and aminopelargonic acid derivatives effectively support growth when added in low concentrations, but not pimelic acid [12]. Biotin auxotrophy of *C. glutamicum *elicits L-glutamate production, a characteristic which led to its discovery. L-Glutamate production by *C. glutamicum *can be triggered in number of alternative ways, e.g. by addition of ethambutol [13] or Tween [14] or by a temperature shift [15].

Triggering L-glutamate production by biotin limitation alters synthesis of fatty acids and mycolic acids [16] as a consequence of reduced activity of acyl-CoA carboxylases, which contain AccBC, one of the two biotin-containing enzymes of *C. glutamicum *[17] as α-subunit. Secretion of L-glutamate is mediated by a carrier [18,19] involving the gene product of *cg1434 *[20], which encodes mechanosensitive channel MscS [21,22]. Activation of MscS without osmotic downshock is thought to result in L-glutamate secretion [20-22].

L-Glutamate production occurs due to reduction of the activity of the tricarboxylic acid cycle enzyme oxoglutarate dehydrogenase (ODHC). The small inhibitory protein OdhI binds to ODHC and inhibits its activity unless it is phosphorylated by serine protein kinase PknG or PknA, PknB and PknL [23-25].

Biotin uptake has not yet been studied in *C. glutamicum*. A sodium-dependent multivitamin transporter and the monocarboxylate transporter 1 are involved in biotin uptake in mammalian cells [26]. A proton symporter is required for biotin uptake in the biotin-auxotrophic yeasts *Saccharomyces cerevisiae *and *Schizosaccharomyces pombe *[27]. In bacteria, several systems for uptake of biotin exist. One biotin uptake system is encoded by the genes *bioM, bioN *and *bioY *and mutations in these genes were shown to result in reduced biotin uptake [28,29]. In bacteria containing only BioY, this protein functions as a high-capacity transporter on its own, while in combination with BioMN it also shows high-affinity towards its substrate biotin [30].

Comparative genome analyses revealed that actinobacteria including *C. glutamicum *possess gene clusters of *bioY, bioM*, and *bioN *and were proposed to import biotin via BioYMN transport systems. In this study, we characterized global gene expression changes due to altered biotin supply and demonstrated that biotin-inducible transport system BioYMN imports biotin.

## Results

### Influence of biotin on global gene expression in wild type *C. glutamicum*

The effect of biotin on global gene expression was studied by transcriptome analysis. Therefore, parallel cultures of *C. glutamicum *WT were grown in CGXII with glucose and either with 1, 200, or 20,000 μg/l biotin (1 μg/l and 20,000 μg/l referred to below as biotin limitation and biotin excess, respectively). RNA was isolated from cells in the exponential growth phase. Relative mRNA levels were then determined by hybridization on whole-genome DNA microarrays [31]. Table 1 shows those genes whose mRNA level was significantly (*P *≤ 0.05) changed by a factor of two or more in three biological replicates in at least one of the comparisons. In response to biotin limitation, 19 genes were differentially expressed with 15 of them showing an increased mRNA level. Upon biotin excess, 20 genes displayed a reduced, one an elevated expression. A comparison of the gene expression changes upon biotin limitation and biotin excess revealed a polar opposite of patterns. The most strongly regulated gene (18.8 fold increase upon biotin limitation, 16 fold decrease upon biotin excess) in this experiment was *cg2147*, which codes for a hypothetical membrane protein with 35% identity to transmembrane protein BioY from *Rhizobium etli*. The two genes downstream of *bioY *(*cg2147*), *cg2148 *and *cg2149*, encoding components of an ABC transport system with 41% and 25% identity, respectively, to ATP-binding protein BioM and energy-coupling factor transporter transmembrane protein BioN from *R. etli*, respectively, also revealed increased mRNA levels under biotin limitation (4.9 and 2.0 fold) and reduced expression upon biotin excess (5.3 and 2.5 fold). The gene *cg2514 *encoding a dipeptide/tripeptide permease showed similar strong expression changes with an mRNA level of 8.9 under limitation and 0.1 upon excess of biotin. Interestingly, two genes of biotin synthesis (*bioA, bioB*) were differentially expressed in response to biotin, as well: 3.8 and 6.8 fold, respectively, increased under biotin limitation and 9.0 and 15.5 fold, respectively, decreased upon biotin excess. The adenosylmethionine-8-amino-7-oxononanoate aminotransferase BioA catalyzes the antepenultimate step of biotin synthesis and biotin synthase BioB catalyzes the final step of biotin synthesis. Thus, expression of genes for a putative biotin uptake system (*bioY, bioM *and *bioN*) and for enzymes of biotin ring assembly (*bioA *and *bioB*) was affected by the biotin availability in the medium. This is in contrast to a previous speculation that not only the capability to synthesize biotin, but also the property to regulate *bio *genes might be lost in *C. glutamicum *[32].

**Table 1:** Gene expression differences of *C. glutamicum *WT in response to biotin limitation, biotin excess or supplementation with dethiobiotin

**Gene**^**a**^	**Annotation**^**a**^	Relative mRNA level		
		**1 μg/l biotin**	**20000 μg/l biotin**	**dethiobiotin**^**b**^
		200 μg/l biotin	200 μg/l biotin	**biotin**^**b**^
*cg0095*	biotin synthase BioB	6.8	0.1	11.3
*cg0096*	hypothetical protein	5.5	0.2	3.6
*cg0097*	hypothetical protein	10.1	0.1	3.5
*cg0126*	hypothetical protein	0.5	n.d.	2.1
*cg0486*	ABC-type transporter. permease component	n.d.	0.5	n.d.
*cg0634*	ribosomal protein L15 RplO	0.4	n.d.	n.d.
*cg1141*	lactam utilization protein	n.d.	0.5	1.2
*cg1142*	transport system	2.1	0.4	1.2
*cg1214*	cysteine desulfhydrase/selenocysteine lyase NadS	1.9	0.5	1.3
*cg1216*	quinolate synthase A NadA	1.9	0.5	1.4
*cg1218*	ADP-ribose pyrophosphatase NdnR	2.1	0.4	2.0
*cg1671*	hypothetical protein	n.d.	2.0	0.3
*cg2147*	Biotin transport protein BioY	18.8	0.1	4.4
*cg2148*	Biotin transport protein BioM	4.9	0.2	2.6
*cg2149*	Biotin transport protein BioN	2.0	0.4	1.6
*cg2320*	predicted transcriptional regulator MarR family	2.0	0.5	1.6
*cg2560*	isocitrate lyase AceA	3.1	0.4	1.0
*cg2747*	metalloendopeptidases-like protein	n.d.	0.4	2.3
*cg2883*	SAM-dependent methyltransferase	2.2	0.2	n.d.
*cg2884*	putative dipeptide/tripeptide permease	8.9	0.1	5.6
*cg2885*	adenosylmethionine-8-amino-7-oxononanoate aminotransferase BioA	3.8	0.1	n.d.
*cg3231*	hypothetical protein	0.5	n.d.	n.d.
*cg3289*	thiol:disulfide interchange protein TlpA	0.4	n.d.	n.d.

^a^Gene numbers and annotations of the revised *C. glutamicum *genome published by NCBI as NC003450

^b^Ratio of the mRNA level in cells grown in CGXII with 200 μg/l dethiobiotin to that of cells grown with 200 μg/l biotin

Dethiobiotin, the substrate of biotin synthase BioB, is the immediate precursor of biotin. To compare global gene expression when *C. glutamicum *is supplemented with dethiobiotin or biotin, parallel cultures of *C. glutamicum *WT were grown in glucose minimal medium with either biotin or dethiobiotin at concentrations of 200 μg/l. In the presence of dethiobiotin, only 9 of the genes listed in Table 1 were differentially expressed, all showing an increased mRNA level similar to those under biotin limitation. The most strongly regulated genes were *bioB*, the gene encoding biotin synthase converting dethiobiotin to biotin (11.3 fold higher than with biotin), *cg2884 *(5.6 fold) and *bioY *(4.4 fold).

### Transcriptional organisation of the putative *bioYMN *operon

**Figure 1:**
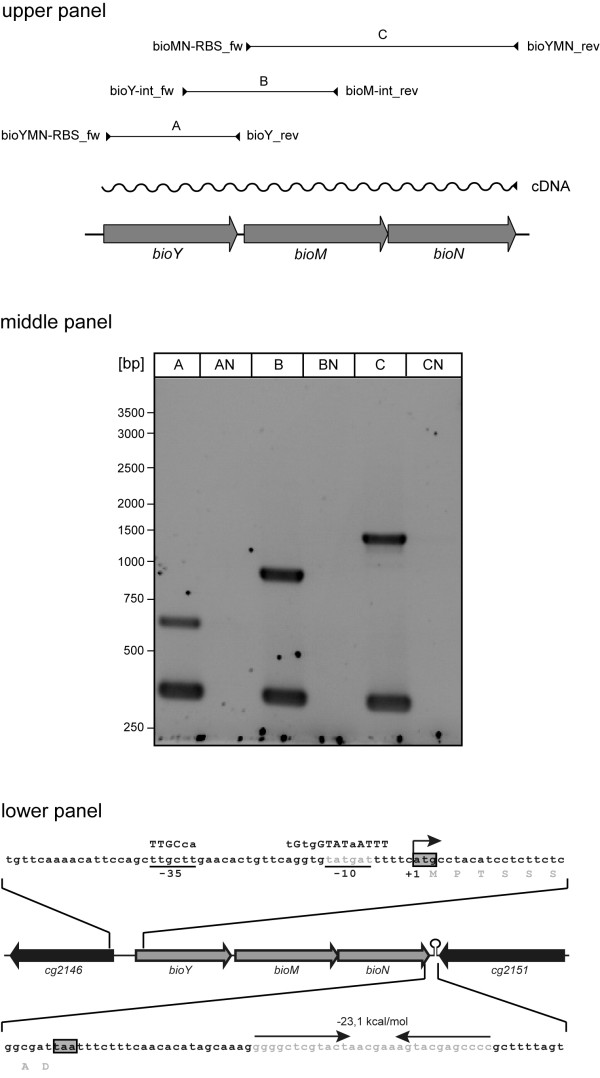
**Transcriptional organization of the *bioYMN *locus in *C. glutamicum***. (upper panel) Scheme showing the *bioYMN *locus in *C. glutamicum *and the RT-PCR reactions used to determine co-transcription of *bioY, bioM *and *bioN*. RNA from *C. glutamicum *WT was transcribed into cDNA with random primers. Subsequently, cDNAs were used as templates for the PCR reactions labeled A-C. (middle panel) Results from the RT-PCR analyses described above. The lower DNA fragment visible lanes A-C represents *dnaE*, and RT-PCR of *dnaE *served as positive control in all reactions. The upper bands in lanes A, B and C correspond to the products of the PCR reactions A-C indicated in A. Reactions AN, BN and CN represent controls confirming the absence of DNA in the RNA preparation. The reactions were identical to the PCR reactions as shown in lanes A-C except that reverse transcriptase was omitted in the cDNA reactions. (lower panel) The *bioYMN *locus is shown schematically. The translational start codon of *bioY *is boxed, the transcriptional start site of *bioYMN *is marked as +1, the -10 and -35 promoter hexamers are given in grey and marked below the *cg2146-bioY *intergenic sequence and are compared to the consensus sequences described in ref. [30], which are depicted above the *cg2146-bioY *intergenic sequence. The translational stop codon of *bioN *and the *bioN*-*cg2151 *intergenic sequence is depicted with a potential transcriptional termination signal rendered in grey and highlighted by arrows above the *bioN*-*cg2151 *intergenic sequence.

As the chromosomal location of *bioY, bioM *and *bioN *and their biotin-dependent gene expression patterns indicated that these genes might form an operon, RT-PCR was applied to test this hypothesis (Figure 1). Total RNA isolated from *C. glutamicum *ATCC 13032 was transcribed into cDNA by using random hexamer primers in a reverse transcriptase reaction. The resulting products were then used for PCR amplifications A to C (Figure 1 upper panel). As shown in the middle panel of Figure 1, cDNA created with random hexamer primers allowed the amplification of a *bioY *fragment (reaction A) and a *bioMN *fragment (reaction C), pointing to an co-transcription of the latter two genes. But further evidence was obtained that *bioYMN *are co-transcribed, since PCR amplification using primers annealing to *bioY *and to *bioM *yielded a PCR product covering the intergenic region and parts of both genes (reaction B). As an internal control in the RT-PCR assays, we used *dnaE *encoding a subunit of DNA polymerase. Besides reactions A, B and C three additional control reactions (AN, BN, CN) were performed; these were identical to reactions A to C, respectively, except that reverse transcriptase was omitted from the initial reactions. The fact that no PCR products were obtained in these reactions confirmed that the RNA was not contaminated with chromosomal DNA.

Since the RT-PCR data indicated that *bioY, bioM *and *bioN *are described as one transcript from one promoter, the RACE-PCR technique was applied to identify transcriptional start sites of *bioY *and *bioM*. Thereby, one transcription start point was identified for the transcription unit *bioYMN *(Figure 1 lower panel), being identical with the first nucleotide (nt) of the *bioY *translational start codon. Comparison of the sequence upstream of the transcriptional start site to the σ^70 ^promoter consensus [33] revealed two hexamers (5'-TTGCTT-3' and 5'-TATGATT-3') which show similarity (9 of 12 identical bases) to the -35 and -10 promoter hexamers and are separated by a spacer of 19 bases (Figure 1 lower panel).

### Characterization of biotin uptake by BioYMN

**Figure 2:**
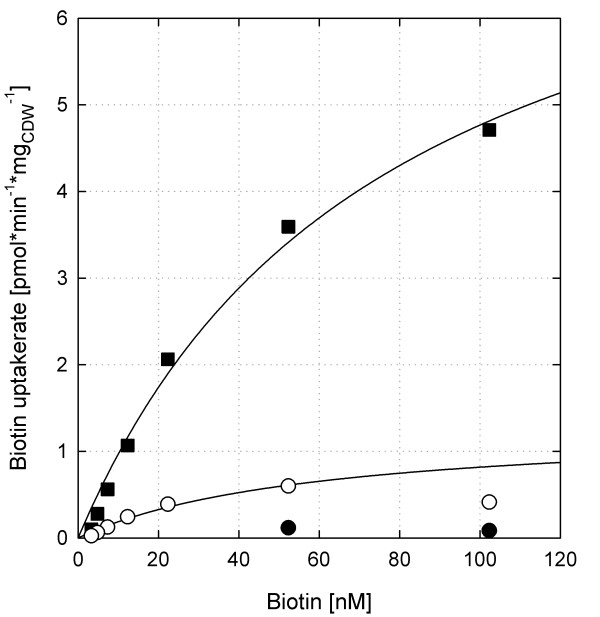
**Biotin transport by *C. glutamicum***. *C. glutamicum *WT(pEKEx3) was grown under biotin-limitation (open circles) or with excess biotin (closed circles) and *C. glutamicum *WT(pEKEx3-*bioYMN*) was grown with excess biotin (closed squares) as described in methods. Uptake rates were plotted as a function of substrate concentration and fitted according to the Michaelis-Menten equation.

In order to demonstrate the direct participation of BioYMN in biotin uptake of *C. glutamicum*, radioactively labelled biotin was used as substrate to determine biotin uptake. For *C. glutamicum *WT(pEKEx3) grown under biotin excess conditions very low transport activities were found (Figure 2). In agreement with the biotin-inducible expression of *bioYMN *(Table 1), significant transport activities were observed for *C. glutamicum *WT(pEKEx3) grown under biotin limiting conditions (Figure 2). In order to characterize the transport activities present under biotin limiting conditions, kinetic parameters were obtained after nonlinear regression according to the Michaelis-Menten equation (Figure 2). Thus, apparent concentrations supporting half-maximal transport rates (*K*_t_) of 60 nM and a maximum rate of transport (*V*_max_) of 1.3 pmol min^-1 ^mg (dry weight)^-1 ^were derived. Due to the very low biotin uptake activities (less than 0.1 pmol min^-1 ^mg (dry weight)^-1^) observed with *C. glutamicum *WT(pEKEx3) grown under biotin excess conditions, the respective kinetic parameters could not be derived. However, the strain overexpressing *bioYMN *under these conditions showed high transport activities with a *K*_t _(77 nM; Figure 2). The *V*_max _of 8.4 pmol min^-1 ^mg (dry weight)^-1 ^determined for *C. glutamicum *WT(pEKEx3-*bioYMN*) grown under biotin excess conditions indicated that biotin uptake rates were at least 50 fold higher when *bioYMN *was overexpressed than in the empty vector control grown under the same conditions.

### Effect of *bioYMN *overexpression on L-glutamate production triggered by biotin-limitation

**Figure 3:**
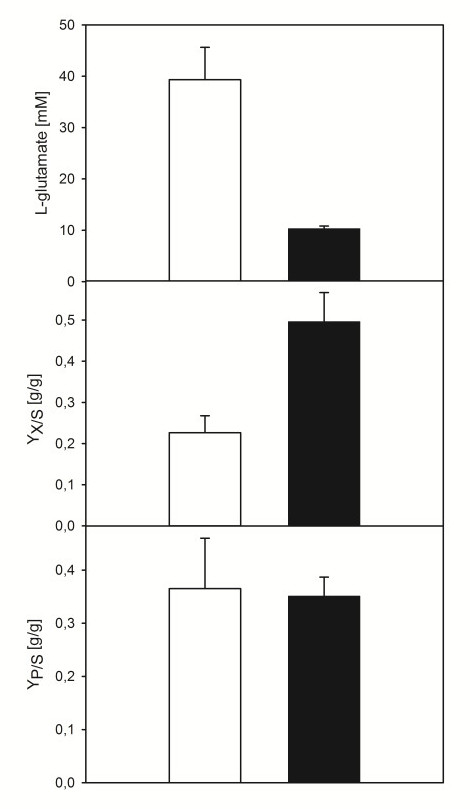
**L-Glutamate production by *C. glutamicum *WT (pEKEx3) (open columns) and WT(pEKEx3-*bioYMN*) (closed columns)**. L-Glutamate concentrations in the culture supernatant (upper panel), biomass yields (g cell dry weight formed per g glucose consumed; middle panel) and product yields (g L-glutamate formed per g glucose consumed) of three fermentations in minimal medium with 40 g/l glucose, 25 μM IPTG and 1 μg/l biotin are given as means with standard deviations.

Biotin limitation triggers L-glutamate production by *C. glutamicum *WT. In order to test if overexpression of *bioYMN *and, thus, overproduction of the concentrative biotin uptake system interferes with triggering L-glutamate production by biotin limitation, biotin-limited precultures of *C. glutamicum *WT(pEKEx3) and WT(pEKEx3-*bioYMN*) were used to inoculate glucose minimal medium cultures with 1 μg/l biotin and 1 mM IPTG and growth and L-glutamate formation was monitored. *C. glutamicum *WT(pEKEx3) accumulated 40 ± 6 mM L-glutamate, formed 3 ± 0.3 g cell dry weight per liter and utilized 88 ± 9 mM glucose (Figure 3 and data not shown). By contrast, WT(pEKEx3-*bioYMN*) formed less L-glutamate (10 ± 1 mM), consumed less glucose (24 ± 2 mM) and formed 1.8 ± 0.1 cell dry weight per liter (Figure 3 and data not shown). While the product yield of both strains was similar (0.36 ± 0.09 and 0.35 ± 0.04 g/g, respectively), WT(pEKEx3-*bioYMN*) showed a higher biomass yield (0.49 ± 0.07 g/g) than the empty vector control (0.23 ± 0.04 g/g; Figure 3). Thus, overproduction of BioYMN alleviated biotin limitation and as a consequence shifted metabolic activity from L-glutamate formation to biomass formation.

## Discussion

Here, we have shown that *C. glutamicum *shows biotin-dependent gene expression changes of the genes encoding the enzymes for biotin ring assembly and for biotin uptake. Moreover, the maximal biotin uptake rate was at least ten fold higher under biotin limitation conditions (1.3 pmol min^-1 ^mg (dry weight)^-1^) as compared to biotin excess conditions (< 0.1 pmol min^-1 ^mg (dry weight)^-1^). These findings are in contrast to the speculation that biotin-auxotrophic *C. glutamicum *has not only lost the ability to synthesize biotin, but also the ability for biotin-dependent gene regulation [32]. BirA of *C. glutamicum *was characterized as monofunctional biotin protein ligase [34] and is not involved in biotin-dependent gene regulation as suggested previously based on bioinformatics analysis [35]. In a similar bioinformatics analysis, a putative transcriptional regulator of the biotin synthesis genes, BioR, has been identified in α-proteobacteria [36]. This GntR-type of transcriptional repressor is encoded together with *bio *genes and putative binding sites named BIOR boxes occur upstream of bio genes and upstream of the regulatory genes in α-proteobacteria [36]. In actinobacteria, TetR-type transcriptional regulators named BioQ were found to be encoded in 11 of 27 genomes as well as a palindromic DNA motif upstream of the *bio *genes. The *bioQ *gene clustered with *bioB *in several genomes of the genera *Nocardia, Rhodococcus, Propionibacterium *and *Mycobacterium *[37]. BioQ is also encoded in the genomes of four *Corynebacterium *species although not clustered with *bio *genes and the predicted BioQ binding sites (TGAAC-N3-GTTCA) occur upstream of the *bio *genes [37]. Although the bioinformatics evidence is convincing, genetic, biochemical or physiologic characterization of this putative transcriptional regulator in actinobacteria has not yet been published.

The biotin-inducible *bioYMN *operon was shown here to encode a functionally active biotin uptake system. BioYMN of *C. glutamicum *likely is essential for survival of this biotin-auxotrophic species as various attempts to delete the operon failed although very high concentrations of biotin were supplemented (data not shown). Restoring biotin prototrophy of *C. glutamicum *has not been reported yet, but it is tempting to speculate that BioYMN is not essential in a biotin-prototrophic recombinant *C. glutamicum *strain. BioYMN from *C. glutamicum *belongs to a type of uptake systems that have been classified as energy-coupling factor (ECF) transporters [38,39]. The core component BioY is active as high-capacity biotin uptake system. In conjunction with the ATP-binding-cassette ATPase BioM and the transmembrane protein BioN, the uptake system shows high affinity for its substrate biotin [30]. *E. coli *cells containing BioY from *R. capsulatus *imported biotin with a *V*_max _of 60 pmol min^-1 ^(mg protein)^-1 ^and a *K*_t _of 250 nM, whereas BioYMN-containing cells exhibited a 50-fold-lower *K*_t _[30]. The *K*_t _of BioYMN from *C. glutamicum *is also in the nanomolar range, but around tenfold lower (60 and 77 nM, respectively, s. above). *C. glutamicum *cells overproducing endogenous BioYMN showed a *V*_max _of 8.4 pmol min^-1 ^(mg protein)^-1^, which is comparable to that determined for *E. coli *cells containing BioYMN from *R. capsulatus *(6 pmol min^-1 ^(mg protein)^-1 ^[30]), but lower than that determined for *E. coli *cells containing only BioY from *R. capsulatus *(60 pmol min^-1 ^(mg protein)^-1 ^[30]).

Amino acid production by the biotin-auxotrophic *C. glutamicum *can be affected positively or negatively by the biotin supply in the medium. Biotin-sufficient conditions are employed for L-lysine production and it has been shown that increasing the biotin supply [40] or overproducing the biotin protein ligase BirA [34] improved L-lysine production. Under biotin-sufficient conditions, the biotin-containing enzyme pyruvate carboxylase is the major anaplerotic enzyme synthesizing oxaloacetate as precursor of L-lysine as deletion of the pyruvate carboxylase gene *pyc *negatively affected L-lysine production [41] whereas deletion of the PEP carboxylase gene *ppc *did not [42]. Accordingly, overexpression of *pyc *improved L-lysine production [41]. On the other hand, L-glutamate production can be triggered by biotin limitation and a role of BioYMN in L-glutamate production by *C. glutamicum *has been found here. Biotin limitation reduces/alters synthesis of fatty and mycolic acids [16] as a consequence of reduced levels of biotinylated AccBC, the α-subunit of the acyl-carboxylases. Moreover, under biotin limitation conditions anaplerosis is not fulfilled by biotin-containing pyruvate carboxylase [41,43], but by PEP carboxylase [44]. In line with the observation that L-glutamate production by *C. glutamicum *wild type is known to be suppressed by an excess of biotin [45], enhancing biotin uptake by overexpression of *bioYMN *decreased L-glutamate production (Figure 3). Thus, BioYMN plays a role in biotin-triggered L-glutamate production by *C. glutamicum*.

## Conclusions

*C. glutamicum *showed biotin-dependent regulation of mRNA levels of *bioA, bioB, bioY, bioM*, and *bioN*. The genes *bioY, bioM*, and *bioN *are transcribed as an operon, *bioYMN*. Transport assays with radio-labeled biotin revealed that BioYMN functions as a biotin uptake system with an affinity for its substrate in the nanomolar range. Overepression of *bioYMN *alleviated biotin limitation and interfered with triggering L-glutamate production by biotin limitation.

## Methods

### Bacterial strains, plasmids, oligonucleotides, and culture conditions

Bacterial strains and plasmids used are listed in Table 2. *Escherichia coli *was grown in lysogeny broth complex medium (LB) as the standard medium [46], while brain heart infusion medium (BHI, Becton Dickinson, Heidelberg, Germany) was used as complex medium for *C. glutamicum*. For growth experiments, in the first preculture, 50 ml BHI medium was inoculated from a fresh BHI agar plate and incubated at 30°C and 120 rpm in baffled flasks. After washing the cells in 0.9% (w/v) NaCl, the second preculture and the main culture were inoculated to an optical density at 600 nm (OD_600_) of 0.5-1.0 in 50 ml CGXII minimal medium [47], which contained 0.03 g/l protocatechuic acid. As carbon and energy sources, 100-250 mM glucose or 200 mM sodium L-lactate were used. Precultures and main cultures were incubated at 30°C and 120 rpm on a rotary shaker in 500 ml-baffled shake flasks. When appropriate, *C. glutamicum *was cultivated with kanamycin (25 μg/ml) or spectinomycin (100 μg/ml). Growth of *C. glutamicum *was followed by measuring the OD_600_. For all cloning purposes, *Escherichia coli *DH5α was used as host.

**Table 2:** Bacteria and plasmids used in this study

Strain, plasmid or oligonucleotide	Relevant characteristics or sequence	Source, reference, or purpose
*E. coli *strains		
DH5α		Culture collection
*C. glutamicum *strains		
ATCC 13032	WT^a^	Culture collection
Plasmids		
pEKEx3	Spec^R^	[48]
pEKEx3-*bioYMN*	pEKEx3 containing *bioY, bioM *and *bioN*	This work
pGEM-T-easy	cloning vector, Amp^R^	Promega
Oligonucleotides		
bio-operon_fw	GATCTAGAATTCAAATTTCAGCCCCCATCC	This work
bio-operon_rev	GATCTAGGATCCCTGACCGCTGGTAACAAG	This work
bioYMN-RBS_fw	AAGGAGATATAGATTTGTTGAACACTGTTCAGGTG	This work
bioMN-RBS_fw	AGGAGATATAGATATGCCCGAGATCATTTTTGACAACAC	This work
bioYMN_rev	TTAATCGCCGGCACCACGTGC	This work
bioY_rev	CTATTTCTTACGGATGTCAGGGAATGC	This work
bioM_rev	TCATTTCGCAGGTTCCGCC	This work
bioY-int_fw	CACAGCGGGAGTGCCTATTGTTTT	This work
bioY-int_rev	GAAGGACGAGACCCACGATG	This work
bioM-int_rev	CAGCGATGATCACTTCTGGCTC	This work
dnaE-fw	TGCCCTTCCGGCGATGTCCAA	[48]
dnaE-rev	CTGGAACCATGTCGTCCTAGAG	[48]

^a ^*WT *wild type

### Determination of amino acid concentrations

During cultivation, samples (1 ml) were collected to determine biomass and amino acid concentrations. The optical density was determined by absorbance at 600 nm. After centrifugation of the sample (13,000 g, 5 min), aliquots of the supernatant were used for analysis. Amino acid concentrations in the culture supernatants were determined by automatic precolumn derivatization with *ortho*-phthaldialdehyde and reversed-phase high-performance liquid chromatography (RP-HPLC) (HP1100 series; Hewlett-Packard, Waldbronn, Germany) with fluorimetric detection (excitation at 230 nm; emission at 450 nm) as described previously [49]. Hypersil ODS 5-mm columns were used (precolumn: 40 × 4 mm; column: 120 × 4 mm, Chromatographie Service GmbH, Langerwehe, Germany). The buffer gradient consisted of 0.1 M sodium acetate, pH 7.2 (with 0.03% sodium azide), as the polar phase and methanol as the nonpolar phase. Quantification was performed with L-asparagine as an internal standard and by comparison with external standards.

### Construction of plasmids and strains

The oligonucleotides listed in Table 2 were obtained from Operon (Cologne, Germany) or MWG (Ebersberg, Germany). Standard methods such as PCR, restriction, and ligation were carried out as described previously [46]. Plasmids were constructed in *Escherichia coli *DH5α from PCR-generated fragments (KOD, Novagen, Darmstadt, Germany) and isolated with the QIAprep spin miniprep kit (QIAGEN, Hilden, Germany). *E. coli *was transformed by the CaCl_2 _method [50], while *C. glutamicum *was transformed via electroporation [51]. All cloned DNA fragments were shown to be correct by sequencing. For homologous overexpression of *bioYMN *the operon was amplified from genomic DNA of *C. glutamicum *WT by using primers bio-operon_fw and and bio-operon_rev, and was sub-cloned to pGEM-T-easy and cloned as 2235 bp-EcoRI-fragment into the expression vector pEKEx3 [48], which allows IPTG-inducible gene expression in *C. glutamicum*.

### Comparative transcriptome analysis using DNA microarrays

Generation of *C. glutamicum *whole-genome DNA microarrays, total RNA preparation, synthesis of fluorescently labelled cDNA, microarray hybridization, washing, and statistical data analysis were performed as described previously [52,53]. Genes exhibiting mRNA levels that were significantly changed (*P *≤ 0.05 in Student's *t *test) by at least a factor of 2.0 were determined in three DNA microarray experiments performed with RNA isolated from three independent cultures.

### RT-PCR analysis and 5'-RACE

RNA was prepared as described previously [52,53] and incubated in 0.1 M sodium acetate, pH 3.0, 5 mM MgSO_4_, and 0.3 U/μl DNase I (Roche Diagnostics, Mannheim, Germany) for 30 min at 37°C. After heat inactivation for 5 min at 75°C, the RNA was precipitated with LiCl as described by [46]. After denaturation for 5 min at 65°C, reverse transcription of 500 ng RNA was performed with Omniscript Reverse Transcriptase (QIAGEN, Hilden, Germany) according to the manufacturer's instructions by using random hexamer primers (Invitrogen, Karlsruhe, Germany). Subsequently, the cDNA was amplified using combinations of the primers A (bioY-RBS_fw, bioY_rev), B (bioY-int_fw, bioM-int_rev) and C (bioMN-RBS_fw, bioYMN_rev). As a control, cDNA of dnaE was amplified using primers RT-dnaE-fw and RT-dnaE-rev. To determine transcriptional starts by RACE-PCR RNA was prepared and purified as described above. Primers binding downstream of the annotated translational starts of *bioY *and *bioM *(bioY_rev, bioM_rev) along with 2.0 μg total RNA were used for cDNA synthesis reverse transcription with Superscript II (Invitrogen, Karlsruhe, Germany) according to the supplier's protocol. After RNA digestion with RNase H (Fermentas, St. Leon-Roth, Germany) and purification the cDNA was then modified by terminal deoxynucleotidyl transferase (Fermentas, St. Leon-Roth, Germany) and dATP respectively dCTP to determinate the transcriptional start accurately. Subsequently, the cDNA was amplified using combinations of oligo(dT) or oligo(dG) primer and either bioY-int_rev or bioM-int_rev. The obtained PCR products were cloned into the pGEM-T Easy vector (Promega, Mannheim, Germany) and transferred into E. coli DH5α cells. At least two different clones per gene were selected for plasmid preparation and DNA sequencing (BigDye Terminator v3.1 Cycle Sequencing Kit and ABI Prism Capillary Sequencer Model 3730, Applied Biosystems, Forster-City, USA).

### Transport assays

Biotin-limited (2.5 μg/l) precultures of *C. glutamicum *WT(pEKEx3) and biotin-sufficient (200 μg/l) precultures of WT(pEKEx3) and WT(pEKEx3-*bioYMN*) were used to inoculate glucose minimal medium cultures with either 1 μg/l or 200 μg/l biotin and allowed to grow to mid-exponential phase in minimal medium CGXII supplied with glucose as the sole carbon source. 1 mM IPTG was used in this culture for 17 h for the induction of pEKEx3-*bioYMN *expression. Subsequently, cells were washed two times with the assay buffer (0.1 M sodium chloride, 25 mM potassium phosphate, pH 7.5) and incubated on ice until the measurement. The cells were energized by incubation for 3 min at 30°C with 20 mM glucose at an optical density (600 nm) of 5 in an assay volume of 2 ml before biotin was added. Finally, 7 kBq of ^3^H-labeled biotin (1.11-2.22 TBq/mmol, PerkinElmer, Rodgau, Germany) was applied in an 2 ml assay at concentrations indicated in the respective experiments, and 200 μl samples were taken at 15, 30, 45, 60, 90 s in order to determine initial uptake rates. Cells were collected on GF55 glass fiber filters (Millipore, Schwalbach, Germany) and washed twice with 2.5 ml of assay buffer. After the resuspension of cells in scintillation fluid (Rotiszinth, Roth, Germany) the radioactivity of the sample was counted in a scintillation counter (Beckman, Krefeld, Germany).

## Authors' contributions

JS and KCS carried out the transcriptional studies, SG, KCS and PPW constructed the recombinant strains and SG and JS performed growth experiments and SM and JS determined the transport activities. RK supervised the transport analyses, participated in the interpretation of the data and critical revision of the manuscript. VFW supervised the experiments and PPW and VFW were responsible for the draft and final version of the manuscript. All authors read and approved the final manuscript.
